# Causality between rheumatoid arthritis and the risk of cognitive impairment: a Mendelian randomization study

**DOI:** 10.1186/s13075-023-03245-x

**Published:** 2024-01-02

**Authors:** Lincheng Duan, Shiyin Li, Haoming Li, Yue Shi, Xiaolong Xie, Yue Feng

**Affiliations:** 1 Chengdu University of Traditional Chinese Medicine, Chengdu, China; 2 Meishan Hospital of Traditional Chinese Medicine, Affiliated Meishan Hospital of Chengdu University of Traditional Chinese Medicine, Meishan, China

**Keywords:** Cognitive impairment, Rheumatoid arthritis, Mendelian randomization

## Abstract

**Background:**

There is mounting proof that rheumatoid arthritis (RA) and cognitive decline are related. These studies, however, have not all been uniform, and others have not discovered such a correlation. It is essential to investigate the link between RA and cognitive decline.

**Method:**

We conducted a Mendelian randomization analysis utilizing three different publicly accessible RA GWAS summary datasets and a variety of meticulously verified instrumental variables. We mostly used inverse variance weighting (IVW), as well as MR-Egger, weighted median, MR-PRESSO, and several sensitivity analyses, to figure out the link between RA and cognitive impairment (CI).

**Results:**

Our MR study identified the causality between RA and declining cognitive performance (*β* = − 0.010, 95% CI of − 0.017 to − 0.003, *P* = 4.33E−03) and cognitive function (*β* = − 0.029, 95% CI of − 0.053 to − 0.005, *P* = 1.93E−02). The consistent direction of the connection is revealed by sensitivity analysis utilizing the weighted median and the MR-Egger method. Furthermore, we reproduced our findings across two additional RA datasets and found identical outcomes, strengthening the validity of our findings.

**Conclusion:**

This study offers proof of causality between RA and an increased risk of CI. Our findings highlight the importance of examining RA patients for cognitive ability, which may open up fresh ideas for the prevention of CI.

**Supplementary Information:**

The online version contains supplementary material available at 10.1186/s13075-023-03245-x.

## Introduction

Rheumatoid arthritis (RA) is an autoimmune disease that affects more than one system [1]. Females and the elderly are more likely to be diagnosed with it, and it affects roughly 1% of the general population [2]. Along with bone degradation and cartilage loss, RA is characterized by peripheral inflammatory polyarthritis [3]. But RA does not just damage the joints; it is also linked to vasculitis, cancer, lung and cardiovascular disease, and mental health problems and, in many instances, can result in lifelong impairment [4]. The prevalence of RA’s extra-articular symptoms varies from 17.8 to 40.9% and can affect a variety of organs, including the nervous and mental systems [5, 6].

Cognitive impairment (CI), a prevalent chronic disorder linked to aging that is characterized by challenges with memory, acquiring new information, problem-solving, concentration, or decision-making, can eventually lead to dementia [7]. Globally, more than 10% of people who are 70 or older have a minor cognitive impairment [8]. However, because some studies have revealed inconsistent results [9–18], the link between RA and CI is still up for debate. Studies have revealed a probable connection between RA and CI, with even moderate CI interfering with everyday functioning in RA patients. While some studies, notably those looking at Alzheimer’s disease and other dementias, have discovered a connection between RA and CI, other studies have not. Largely unclear are the processes that underlie the association between RA and cognitive decline. Chronic inflammatory conditions [19, 20], immune system modifications [21], and ongoing pain and discomfort [22–24] are possible explanations. Mechanistic research should be scientifically developed and put into practice in the future since it is not yet sufficient to support any of these mechanisms.

Therefore, the relationship between RA and the emergence of CI is still unclear. Due to confounding bias, association inference from this earlier observational data may be constrained, and even findings may have reverse causality. Randomized controlled trials (RCTs) can show that RA and CI are causally related, but they are costly and time-consuming. Mendelian randomization (MR), a new method, examines whether observed correlations between exposure variables and outcomes are compatible with causal effects by utilizing genetic variation as an instrumental variable (IV) [25]. Confounding variables and reverse causal linkages may be avoided for correlation effects, and bias can be minimized since genetic variation is unaffected by external environmental, social, behavioral, or other factors [26]. Therefore, the goal of this study was to determine the causative link between RA and CI using a two-sample MR analysis.

## Methods

### Study design

**Fig. 1:**
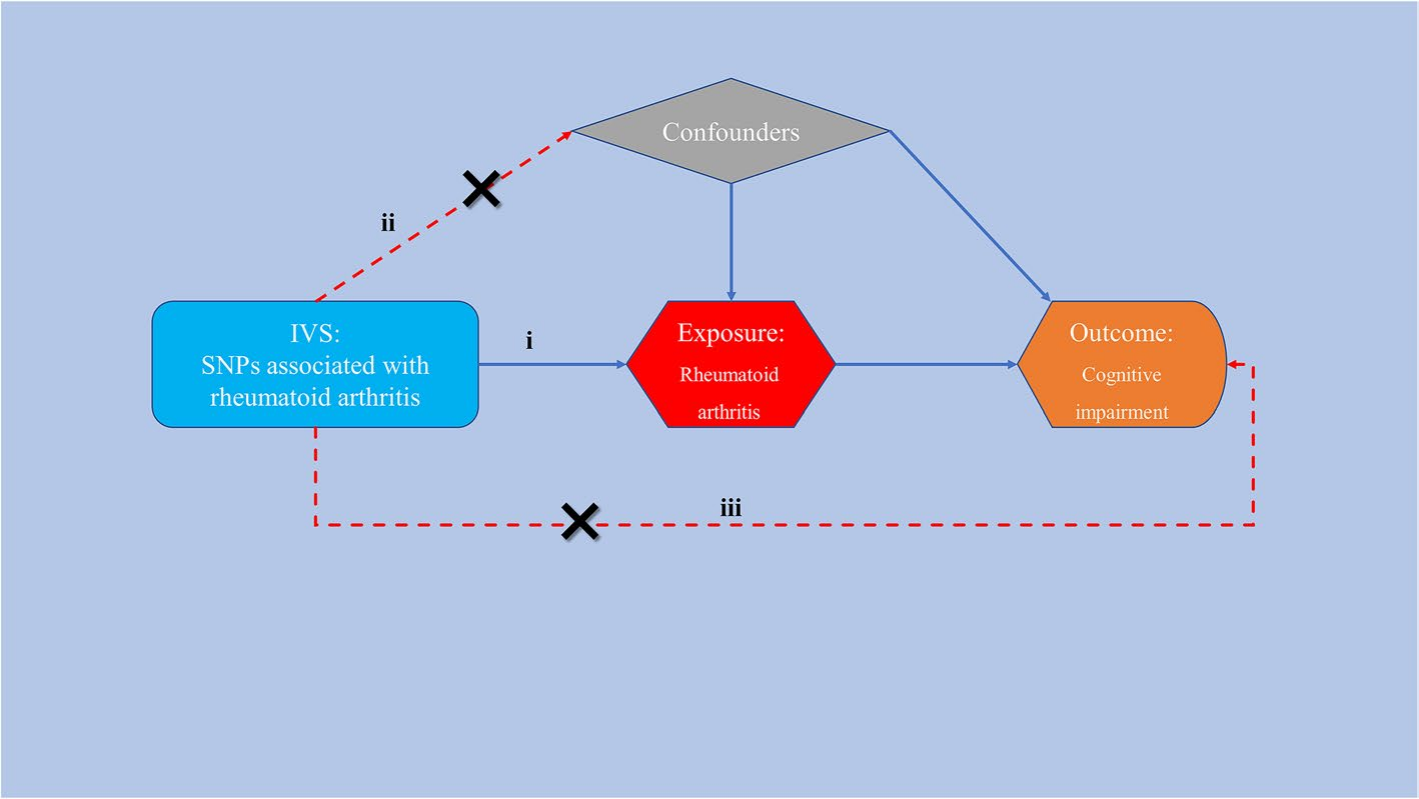
Study design for our MR study

The largest publicly accessible GWAS datasets were used in our two-sample MR analysis to investigate the causal link between RA and the risk of cognitive impairment. Three critical assumptions (Fig. 1) must be met by the effective instrumental variables (IVs) during the MR analysis process in order to obtain trustworthy results [27]: (i) the IVs and RA exhibit a strong correlation, (ii) there is no connection between the IVs and any confounding variables that might affect both RA and CI, and (iii) IVs cannot affect CI by any other means besides RA, but only via RA. The most recent recommendations (STROBE-MR) were followed in this investigation [28].

### Data sources

We used publicly accessible summary-level GWAS data; specifics are presented in Table 1. Specifically, a total of 74,823 controls and 22,350 RA patients made up the most recent summary dataset for this disease as the discovery sample [29], obtained from the GWAS catalog (https://www.ebi.ac.uk/gwas/). As replication samples, we also employed two additional RA datasets [30, 31] from the IEU OpenGWAS database (https://gwas.mrcieu.ac.uk/datasets/). For outcomes, the Within Family GWAS Consortium, which included 22,593 samples, provided the summary data on cognitive function [32]. A general cognitive function score was used to evaluate cognitive function; higher scores corresponded to greater cognitive functioning, and vice versa. Comprised of 257,841 participants of European ancestry, the biggest publicly available GWAS dataset offers a summary of information on cognitive performance [33]. It combines recently released findings from the COGENT consortium with earlier findings from a UKB investigation of cognitive ability. Finally, the features of exposure and outcome had no sample overlap, and all study participants were of European descent.

**Table 1:** The GWAS data source details in our study

Phenotype	Data source	Consortium	PMID	Sample size	Ancestry
Rheumatoid arthritis	GWAS catalog	NA	36333501	97,173	European
Rheumatoid arthritis	IEU OpenGWAS	NA	33310728	58,284	European
Rheumatoid arthritis	IEU OpenGWAS	NA	23143596	47,580	European
Cognitive performance	IEU OpenGWAS	SSGAC	30038396	257,841	European
Cognitive function	IEU OpenGWAS	Within family GWAS consortium	35534559	22,593	European

### Instrumental selection

From the related GWAS pooled datasets, we first identified SNPs that were strongly linked with RA (*P* < 5.00E−08). We next used a reference set of 1000 genomes from a European population to rule out linkage disequilibrium between these SNPs (*r*^2^ < 0.001 and clump window > 10,000 kb). Palindromic SNPs were also manually eliminated. Following these procedures, the SNPs that were left over were utilized as instrument variables. To reduce weak instrumental bias (*F* > 10), we also employed the *F*-statistic (*F* = *β*^2^/se^2^) to determine the statistical efficacy of SNPs and to exclude SNPs with poor statistical efficacy [34].

### Mendelian randomization analysis

We employed the IVW approach in our MR study as the main analysis, adding MR-Egger, weighted median, and MR-PRESSO as supplements and sensitivity analyses. While assuring that all IVs are valid, IVW can provide the most accurate assessment of causality [35]. Although not very efficient, the MR-Egger regression technique may yield estimates with a pleiotropy adjustment, and its intercept can be used to check if pleiotropy is present or not [36]. If half of the instrumental weights are derived from reliable instrumental variables, the weighted median method will yield unbiased causal estimates [37].

### Sensitivity analysis

Further, several sensitivity analyses were carried out to evaluate the results’ robustness. First, we used Cochran’s *Q* test to measure the heterogeneity of the IVW, and funnel plots were used to display the findings. Additionally, we used radial_IVW to find and remove IVs that contribute more to heterogeneity [38]. Pleiotropy was examined using the MR-Egger intercept test, and the outcomes were shown using scatterplots. The MR-PRESSO test is also used to test for horizontal pleiotropy, identify outliers, and offer causal estimates when the corresponding outliers have been eliminated [39]. Furthermore, we carried out a leave-one-out analysis to see if a single SNP significantly changed the outcomes. In the end, we performed an MR-Steiger analysis to check the validity of our causal directions. The “TwoSampleMR” (version 0.5.6), “Radial MR” (version 1.0), and “MRPRESSO” (version 1.0) packages, together with RStudio (version 4.2.1), were used to conduct the whole analysis.

## Results

**Fig. 2:**
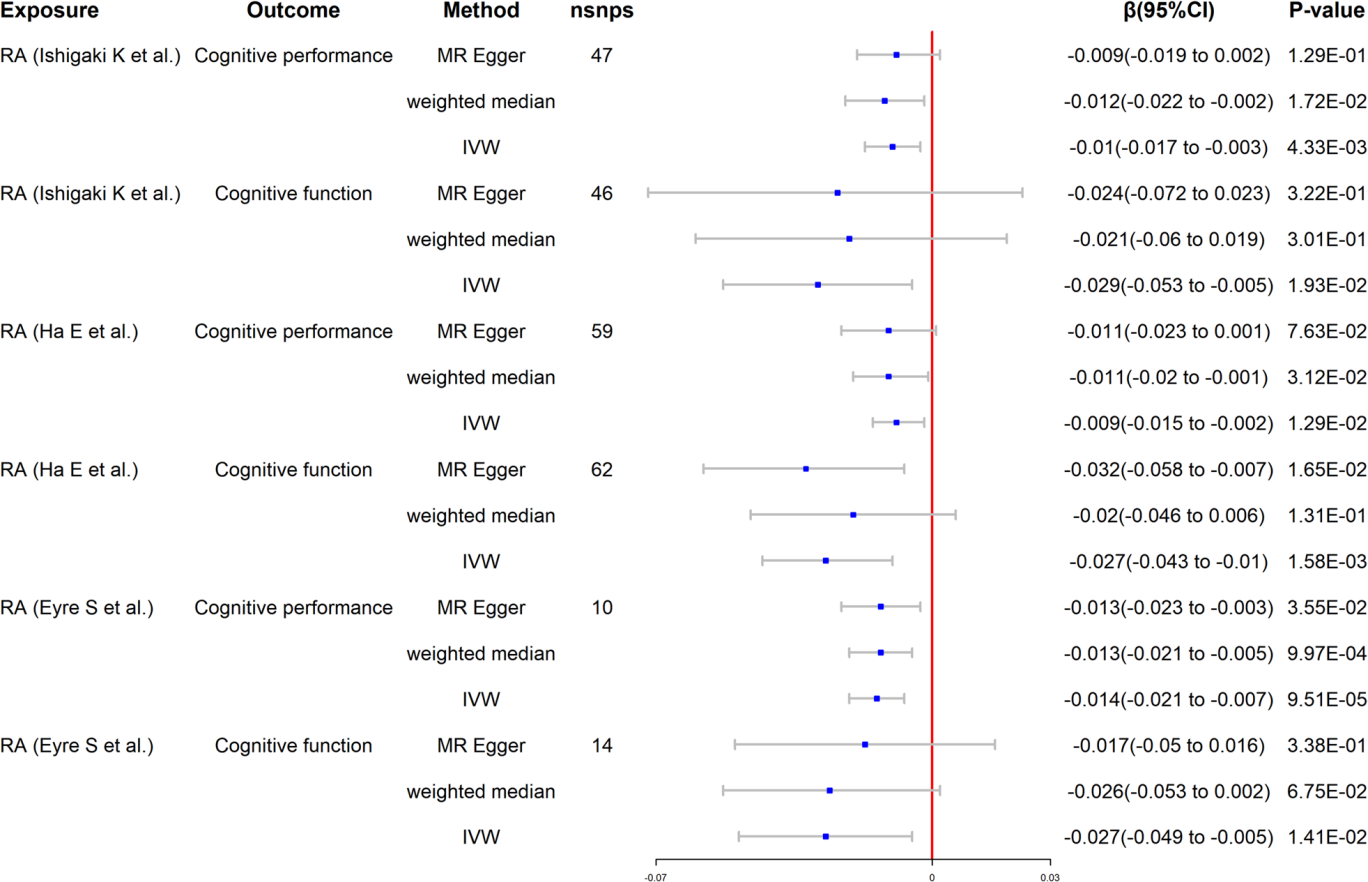
Mendelian randomization for the association of RA on CI

Following thorough screening, we determined 46 and 47 SNPs to be IVs for the outcomes of cognitive performance and cognitive function, respectively (Additional file 2: Table S1). There is no mild instrumental bias, as shown by the *F*-statistics, which are all bigger than 10. The MR results of RA on CI are listed in Fig. 2.

We employed a fixed-effects model using the IVW technique since Cochran’s *Q* test did not reveal heterogeneity (*P* > 0.05). Specifically, using the IVW approach, we found significant associations between RA and the risk of cognitive performance (*β* = − 0.010, 95% CI of − 0.017 to − 0.003, *P* = 4.33E−03) and cognitive function (*β* = − 0.029, 95% CI of − 0.053 to − 0.005, *P* = 1.93E−02) decline. The outcomes of the weighted median, MR-PRESSO, and MR-Egger analyses were similar to those of the IVW approach (Fig. 2). Additionally, we achieved identical results in duplicated samples, demonstrating the validity of the findings. Furthermore, we did not detect heterogeneity or pleiotropy of effects in our investigation using the MR-Egger intercept and Cochran’s *Q* test (Table 2). The MR-Steiger test also validates the correctness of all of our causal directions (Table 3).

**Table 2:** Sensitivity analysis of the associations between RA and CI

Exposures	Outcomes	Heterogeneity test				Pleiotropy test		MR-PRESSO
		MR-Egger		IVW		MR-Egger intercept		Global test
		*Q*	pval	*Q*	pval	Intercept	*P*	*P*
**Sensitivity analysis in the discovery samples**								
RA (Ishigaki et al.)	Cognitive performance	52.06	2.18E−01	52.19	2.46E−01	− 2.84E−04	7.36E−01	2.56E−01
RA (Ishigaki et al.)	Cognitive function	27.61	9.75E−01	27.66	9.80E−01	− 6.41E−04	8.32E−01	9.75E−01
**Sensitivity analysis in replication samples**								
RA (Ha et al.)	Cognitive performance	62.69	2.82E−01	62.99	3.04E−01	4.50E−04	6.03E−01	3.30E−01
RA (Ha et al.)	Cognitive function	51.32	7.80E−01	51.64	7.98E−01	1.20E−03	5.73E−01	8.11E−01
RA (Eyre et al.)	Cognitive performance	8.38	3.97E−01	8.42	4.92E−01	− 3.11E−04	8.44E−01	6.23E−01
RA (Eyre et al.)	Cognitive function	6.03	9.14E−01	6.68	9.18E−01	− 3.56E−03	4.36E−01	9.46E−01

**Table 3:** Results of MR Steiger direction test

Exposure	Outcome	snp_r2. exposure	snp_r2. outcome	correct_causal_direction	steiger_pval
RA (Ishigaki et al.)	Cognitive performance	4.80E−02	2.38E−04	True	0
RA (Ishigaki et al.)	Cognitive function	3.67E−02	1.54E−03	True	6.34E−95
RA (Ha et al.)	Cognitive performance	8.30E−02	2.70E−04	True	0
RA (Ha et al.)	Cognitive function	1.15E−01	2.94E−03	True	4.65E−306
RA (Eyre et al.)	Cognitive performance	3.18E−02	9.16E−05	True	1.49E−256
RA (Eyre et al.)	Cognitive function	4.25E−02	5.87E−04	True	2.38E−111

## Discussion

This study is the first that we are aware of to evaluate the causal link between RA and CI using MR technology. The studies’ findings suggested that RA was associated with an increased risk of CI. Our work emphasizes the significance of understanding the effect of RA on cognitive function and addressing it in the treatment of the illness.

Numerous earlier studies have also shown that RA patients have a considerably elevated risk of cognitive impairment, which is consistent with our findings. Meade et al. [10] carried out a systematic review of CI in RA patients. They came to the conclusion that, when compared to healthy controls, patients with RA performed badly on cognitive functioning tests, notably in the areas of verbal performance, memory, and focus. Our findings are in line with data from a meta-analysis of 16 trials that show RA patients are at risk for CI [11]. A study conducted on a cohort of 1449 individuals demonstrated a strong correlation between the occurrence of any joint ailment during midlife and subsequent cognitive decline over a period of 21 years [40]. Similar findings were seen in Thailand among 464 patients with RA [41], where higher RA disease activity was linked to a higher likelihood of CI, in support of another cross-sectional study [42]. Another prospective study found that those with arthritis had poorer cognitive functioning than those without it, as measured by lower scores on situational memory, mental status, and general cognition [43]. However, other studies have found that RA is not associated with CI [13–17] or even shows a negative correlation [18, 44, 45]. Two other studies using data from the US Health and Retirement Study (HRS) did not find an association between RA and CI13, 15]. A Korean nested case-control investigation also neglected to find a link between RA and dementia [17], even as another case-control study suggested a negative association between RA and AD [45]. Also, a Taiwanese case-control study found that people with RA were 37% less likely to get dementia than people without RA, and the risk was even lower for people with RA who were taking antirheumatic drugs [44], the same conclusions as those obtained in another cohort study [18]. Additionally, two other MR studies conducted recently have produced contradictory results about the causal link between RA and AD [46, 47].

Currently, there is limited understanding of the factors that contribute to the association between RA and CI. Potential processes that could be implicated include the presence of chronic inflammation, participation of the immune system, adverse effects of medications, genetic variables, and the presence of persistent pain and psychiatric illnesses. First, by analyzing the relationship between cognitive performance and peripheral lymphocyte subsets in RA patients, Petersen et al. [48] demonstrated that the linkage between RA and cognitive impairment may be due to premature immunological senescence. A number of autoantibodies and brain-derived proteins, such as anti-myelinating oligodendrocyte glycoprotein (MOG) IgG and S100 calcium-binding beta (S100), may also be linked to cognitive impairment in RA [49–51]. Cognitive decline may result from these causes, which may have a negative impact on the number of neurons and synapses as well as the rate of information processing. Additionally, chronic inflammation with high levels of chemokines (CXCL10, CXCR3) [21, 52] and pro-inflammatory cytokines (IL-1, IL-6, and TNF−) [21, 53, 54] has been identified as a significant contributor to CI. Furthermore, some regularly used RA medications (such as methotrexate and corticosteroids) may be linked to CI in RA patients [10, 12, 19, 55]. While the mechanisms that contribute to the association between RA and CI remain uncertain, it is recommended in professional practice to implement preventive strategies, such as regular evaluation of cognitive function in individuals diagnosed with RA.

The construction of MR involves the utilization of openly accessible GWAS summary statistics. It is feasible to mine trustworthy genetic data without incurring additional experimental expenses. Secondly, we confirmed our findings with other data sets, which increased the credibility of our conclusions. Additionally, a series of sensitivity studies were conducted to showcase the robustness of the findings.

There are also some limitations to our study. First, due to the limits of the data, stratification by age or sex was not done. Second, the exposure and outcome analyses limited the research population to Europeans alone. To what extent these findings hold true for other populations needs to be investigated. Significant GWAS data must support the study and include participants from other ethnic groups. Finally, due to restrictions in the data sources, we were unable to investigate other different subtypes of cognitive impairment. For future analysis, larger, more thorough datasets might be required.

## Conclusion

In conclusion, the present study demonstrated a significantly increased risk of CI in RA patients. It emphasizes the need to monitor cognitive function in RA patients, and further studies on the mechanisms of the role between the two are needed in the future.

### Supplementary Information

**Additional file 1:** **Fig. S1.** MR plots for the causal association of RA (Ishigaki K et al.) on cognitive performance. (A) Forest plot: each dot and its corresponding line represent the effect size and 95%CI. Each dot and its corresponding line represent the pooled estimates after the removal of the corresponding SNP. (B)Funnel plot: the x-axis represents β, and the y-axis represents 1/SE (standard error). (C) The leave-one-out sensitivity analysis: each dot and its corresponding line represent the pooled estimates after the removal of corresponding SNP. (D) Scatter plots: the estimate of intercept can be interpreted as an estimate of the average pleiotropy of all single-nucleotide polymorphisms (SNPs), and the slope coefficient provides an estimate of the bias of the causal effect. **Fig. S2.** MR plots for the causal association of RA (Ishigaki K et al.) on cognitive function. (A) Forest plot. (B) Funnel plot. (C) The leave-one-out sensitivity analysis. (D) Scatter plot. **Fig. S3.** MR plots for the causal association of RA (Ha E et al.) on cognitive performance. (A) Forest plot. (B) Funnel plot. (C) The leave-one-out sensitivity analysis. (D) Scatter plot. **Fig. S4.** MR plots for the causal association of RA (Ha E et al.) on cognitive function. (A) Forest plot. (B) Funnel plot. (C) The leave-one-out sensitivity analysis. (D) Scatter plot. **Fig. S5.** MR plots for the causal association of RA (Ha E et al.) on cognitive performance. (A) Forest plot. (B) Funnel plot. (C) The leave-one-out sensitivity analysis. (D) Scatter plot. **Fig. S6.** MR plots for the causal association of RA (Ha E et al.) on cognitive function. (A) Forest plot. (B) Funnel plot. (C) The leave-one-out sensitivity analysis. (D) Scatter plot.

** Additional file 2:** **Table S1.** Detailed information of instrumental variables used in the MR analysis.

## Data Availability

The datasets used and/or analyzed during the current study are available from the corresponding authors upon reasonable request.
