# Supplementary material for: Causality between rheumatoid arthritis and the risk of cognitive impairment: a Mendelian randomization study
Source: Arthritis Res Ther. 2024 Jan 2;26:5. doi: 10.1186/s13075-023-03245-x (PMC10759661; doi:10.1186/s13075-023-03245-x)
Supplement: Supplementary file 1 — Additional file 1: Fig. S1. MR plots for the causal association of RA (Ishigaki K et al.) on cognitive performance. (A) Forest plot: each dot and its corresponding line represent the effect size and 95%CI. Each dot and its corresponding line represent the pooled estimates after the removal of the corresponding SNP. (B)Funnel plot: the x-axis represents β, and the y-axis represents 1/SE (standard error). (C) The leave-one-out sensitivity analysis: each dot and its corresponding line represent the pooled estimates after the removal of corresponding SNP. (D) Scatter plots: the estimate of intercept can be interpreted as an estimate of the average pleiotropy of all single-nucleotide polymorphisms (SNPs), and the slope coefficient provides an estimate of the bias of the causal effect. Fig. S2. MR plots for the causal association of RA (Ishigaki K et al.) on cognitive function. (A) Forest plot. (B) Funnel plot. (C) The leave-one-out sensitivity analysis. (D) Scatter plot. Fig. S3. MR plots for the causal association of RA (Ha E et al.) on cognitive performance. (A) Forest plot. (B) Funnel plot. (C) The leave-one-out sensitivity analysis. (D) Scatter plot. Fig. S4. MR plots for the causal association of RA (Ha E et al.) on cognitive function. (A) Forest plot. (B) Funnel plot. (C) The leave-one-out sensitivity analysis. (D) Scatter plot. Fig. S5. MR plots for the causal association of RA (Ha E et al.) on cognitive performance. (A) Forest plot. (B) Funnel plot. (C) The leave-one-out sensitivity analysis. (D) Scatter plot. Fig. S6. MR plots for the causal association of RA (Ha E et al.) on cognitive function. (A) Forest plot. (B) Funnel plot. (C) The leave-one-out sensitivity analysis. (D) Scatter plot. [file 13075_2023_3245_MOESM1_ESM.docx]

**Causality between rheumatoid arthritis and the risk of cognitive impairment: a Mendelian randomization study**


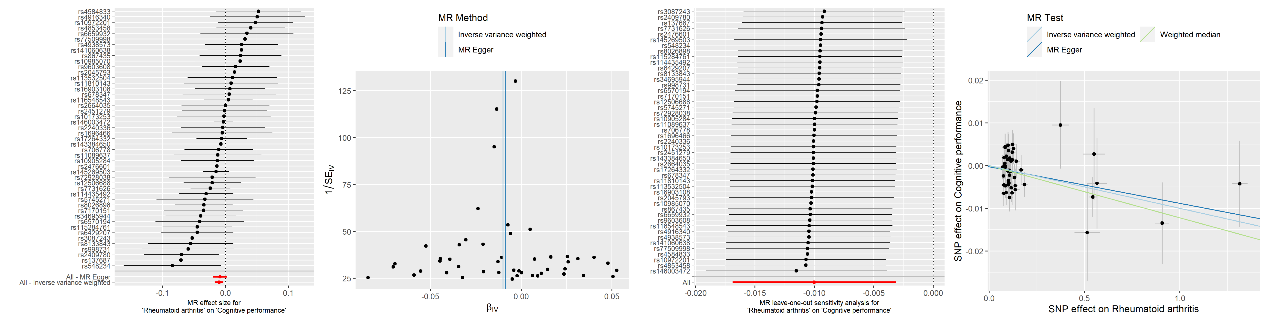


**Supplementary Figure 1.** MR plots for the causal association of RA (Ishigaki K et al.) on cognitive performance. (A) Forest plot: each dot and its corresponding line represent the effect size and 95%CI. Each dot and its corresponding line represent the pooled estimates after the removal of the corresponding SNP. (B)Funnel plot: the x-axis represents β, and the y-axis represents 1/SE (standard error). (C) The leave-one-out sensitivity analysis: each dot and its corresponding line represent the pooled estimates after the removal of corresponding SNP. (D) Scatter plots: the estimate of intercept can be interpreted as an estimate of the average pleiotropy of all single-nucleotide polymorphisms (SNPs), and the slope coefficient provides an estimate of the bias of the causal effect.


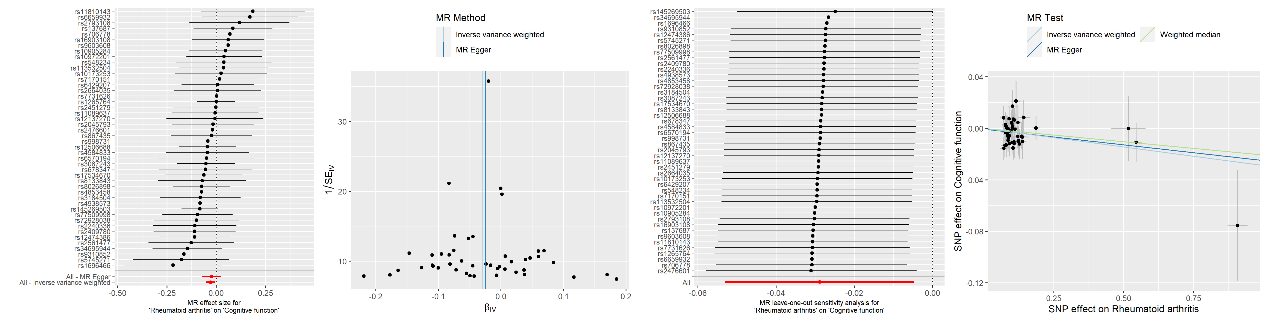


**Supplementary Figure 2.** MR plots for the causal association of RA (Ishigaki K et al.) on cognitive function. (A) Forest plot. (B) Funnel plot. (C) The leave-one-out sensitivity analysis. (D) Scatter plot.


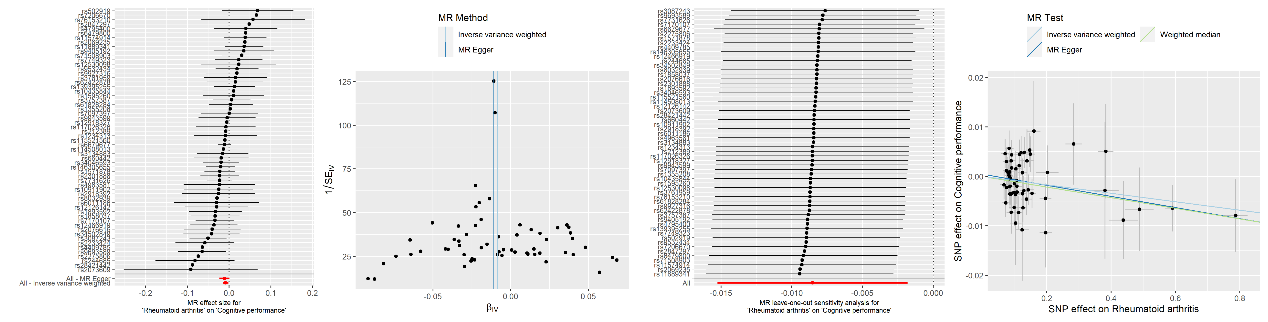


**Supplementary Figure 3.** MR plots for the causal association of RA (Ha E et al.) on cognitive performance. (A) Forest plot. (B) Funnel plot. (C) The leave-one-out sensitivity analysis. (D) Scatter plot.


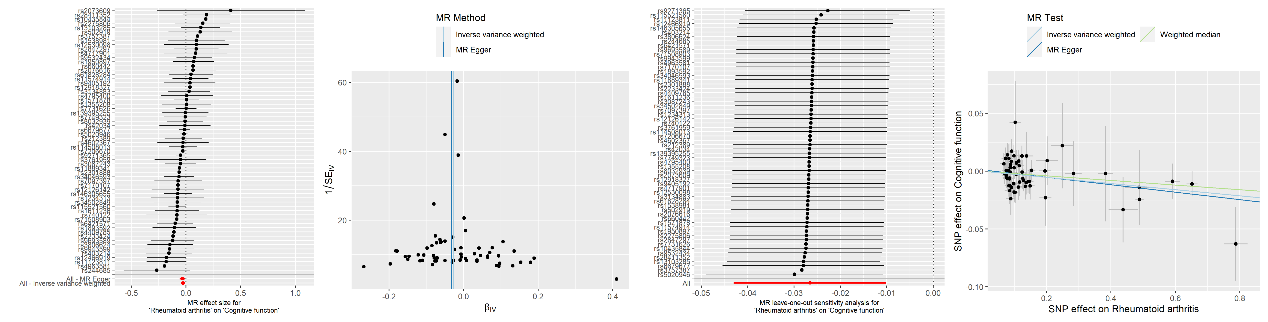


**Supplementary Figure 4.** MR plots for the causal association of RA (Ha E et al.) on cognitive function. (A) Forest plot. (B) Funnel plot. (C) The leave-one-out sensitivity analysis. (D) Scatter plot.


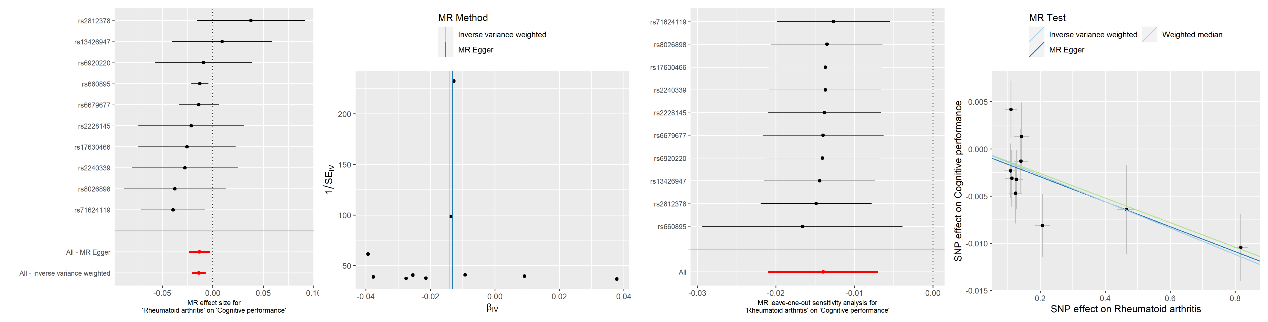


**Supplementary Figure 5.** MR plots for the causal association of RA (Ha E et al.) on cognitive performance. (A) Forest plot. (B) Funnel plot. (C) The leave-one-out sensitivity analysis. (D) Scatter plot.


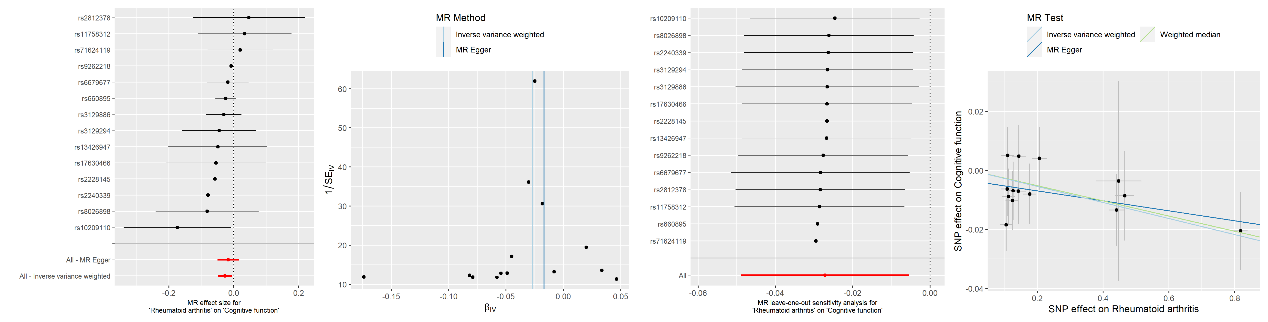


**Supplementary Figure 6.** MR plots for the causal association of RA (Ha E et al.) on cognitive function. (A) Forest plot. (B) Funnel plot. (C) The leave-one-out sensitivity analysis. (D) Scatter plot.
